# Supplementary material for: Content-rich biological network constructed by mining PubMed abstracts
Source: BMC Bioinformatics. 2004 Oct 8;5:147. doi: 10.1186/1471-2105-5-147 (PMC528731; doi:10.1186/1471-2105-5-147)
Supplement: Additional File 2 — The original results of the above study (non-essential files are deleted to keep the file size under the limit set by BMC bioinformatics). [file 1471-2105-5-147-S2.bz2 › chilibotAdditionalFile2/dip10/743ID9393690E18122/html/OMPF_TOLA.html]

 


 **OMPF** and **TOLA** 
  
Found 9 abstracts in PubMed, retrieved 9.  
 

 What does Google say? 
 PDF only 
| .edu only 

---

**Interactive relationship** (e.g. stimulation, inhibition, etc)

**Neutral relationship**- **TolA**  interacted with  **OmpF** , PhoE and LamB porins via its central domain, but not with either their denatured monomeric forms or OmpA.  Ref: 8978668 EMBO J, 1996
- Western blot analyses of cell extracts with anti  **TolA**  antibodies indicated that  **TolA**  forms high molecular weight complexes specifically with trimeric  **OmpF** , OmpC, PhoE and LamB, but not with OmpA.  Ref: 8978668 EMBO J, 1996

**Non-interactive relationship** (e.g. studied together, co-existance, homology, etc.)

- In this study, we demonstrate that the genes tolQ, tolR, and  **tolA** , but not tolB, tolC, and  **ompF** , are required for the internalization of cloacin DF13 and they are not involved in the transport of ferric aerobactin.  Ref: 8419302 J Bacteriol, 1993
- In mixtures of P domain and  **TolA**  with  **OmpF**  porin, only binary and no ternary complexes were observed, suggesting that binding of these proteins to the porin is mutually exclusive.  Ref: 10913271 Biochemistry, 2000
- Furthermore,  **tolA**  876 staA 2 and  **tolA**  staA 2 mutants were 10 to 2700 times more resistant than staA strains to bacteriophages TuIa, TuIb and T4, and TuII whose receptors are major outer membrane proteins  **OmpF** , OmpC and OmpA, respectively.  Ref: 2040438 FEMS Microbiol Lett, 1991
- Bacteriocin sensitivity tests performed on a collection of E. coli envelope mutants, and isolation and characterization of E. coli bacteriocin 28b insensitive mutants, showed that the core lipopolysaccharide, outer membrane proteins OmpA and  **OmpF** , and TolQ,  **TolA** , and TolB proteins are involved in bacteriocin 28b lethal activity.  Ref: 8595593 Can J Microbiol, 1996
- We consider a variety of different colicins, including A, B, D, E1, E3, Ia, M and N, that penetrate through the porins  **OmpF** , FepA, BtuB, Cir and FhuA, to subsequently interact with a few targets in the periplasm, including  **TolA** , TolB, TolC and TonB.  Ref: 12423783 Biochimie< MedlineTA>Biochimie, 1996
- It then uses  **OmpF**  to translocate across the outer membrane and in the process it also needs domains II and III of the protein  **TolA** .  Ref: 10913271 Biochemistry, 2000
